# Glances at the Hospitals

**Published:** 1903-08-22

**Authors:** 


### GLANCES AT THE HOSPITALS.

### THE CLAYTON HOSPITAL, WAKEFIELD.

The total ordinary income amounted to £5,411, and the total ordinary expenditure to £4,328. The daily average number of beds occupied was 55. The death-rate was 7·44 per cent., but deducting 15 cases dying within 48 hours of admission, the percentage is reduced to 5·2. The average cost of each in-patient was £5 10s. 10d., and the cost per bed occupied was £66 19s. 8d. The cost of each out-patient was 3s. 2d. At the beginning of the year there were 52 patients in the hospital and 620 were admitted during the twelve months, making a total of 672 under treatment. Of these 436 were discharged cured, 123 were discharged relieved, 13 were discharged not improved, 50 died, and 50 were left in the hospital. The medical tables are arranged so that columns show the numbers "cured," "relieved," "not relieved," and "died." Of 11 cases of acute rheumatism 9 were cured and 2 relieved. Of 6 cases of pneumonia 5 were cured and 1 died. Similar particulars are given of the surgical cases, and it is therefore curious that the Operation Table does not show results, nor is there any table showing what anæsthetics were used in the 229 operations.

### SOUTHPORT INFIRMARY.

The ordinary income was £3,982, and the ordinary expenditure £4,055. There were 57 patients in residence at the beginning of the year, and 601 were admitted during it, making a total of 658 under treatment. The average number of beds occupied was 52, and the average duration of each patient's stay in the hospital was 29 days. Of the total number, 396 were discharged cured; 108 were discharged relieved; 14 were discharged not improved; 41 for other reasons; and 47 died. The number remaining was 52. There are no proper medical or surgical tables; except one which shows the causes of death. The others only give numbers and might be as well omitted altogether.

### THE BLACKBURN INFIRMARY.

It is pleasing to note that the funds of this hospital are in a satisfactory condition. The total income from all sources amounted to £7,160, and the expenditure to £6,670. The number of in-patients during the year was 1,496, of which 73 remained from the previous year. The average stay in the hospital was 26 days, and the average number resident was 89. There were 82 deaths, being at the rate of 6·5 per cent., but if 19 be excluded as dying within 48 hours of admission, the death-rate would be only 5 per cent. We did not notice any statement of the cost per patient, or of the cost per bed occupied, omissions which we hope will be rectified in the next annual report. Of the total number of in-patients, 1,168 were discharged cured or relieved. The medical and surgical tables are clearly drawn out. Of 20 cases of acute rheumatism all were cured. The death-rate from pneumonia was high, namely, 3 out of a total of 8. Doubtless there is a satisfactory explanation of this; and if the tables had a fourth column for "remarks," such explanation could be easily given. The operation table tells us that ovariotomy was performed three times, all being successful, and the same result followed in 13 operations for the radical cure of hernia. On the other hand, of four operations for perforating gastric ulcer, all died, and here again, we think, the fourth column for "remarks" would have been interesting. The total number of operations was 395, and there were only 19 deaths. There is no table showing the nature of the anæsthetics used. As the medical report is carefully prepared as far as it goes, it seemed a pity that information on the points referred to is not supplied.

### HOSPITAL FOR SICK CHILDREN, PLAISTOW, LONDON.

The ordinary income was £3,254, and the ordinary expenditure was £3,324, showing a deficit of about £70, but £105 was expended on drainage, so that so far as maintenance goes there was a small balance on the right side. It is proposed to extend the hospital and for this purpose at least £23,000 will be required. The total number of in-patients was 453. Of these 316 were cured, 46 relieved, 10 not relieved, 65 died, and 22 remained in the hospital. Of 8 cases of enteric fever 7 recovered and 1 was under treatment. Of 19 cases of acute bronchitis 18 recovered and 1 died. Of 28 cases of broncho-pneumonia 12 recovered and 16 died, a mortality which shows what a serious disease this is in children. As will be seen from the above extracts, the medical and ordinary surgical tables are properly compiled, but when we come to the operations we find that only numbers are given. It is not of much use to learn that there were 8 cases of resection of rib if we are told nothing of the results. Altogether there were 664 operations performed under anæsthetics. The A.C.E. mixture was given 433 times, chloroform 122 times, ether once, nitrous oxide 51 times, cocaine 35 times, beta-eucaine 21 times, and A.C.E. mixture followed by ether once.

